# Variability and dimensionality of students’ and supervisors’ mini-CEX scores in undergraduate medical clerkships – a multilevel factor analysis

**DOI:** 10.1186/s12909-018-1207-1

**Published:** 2018-05-08

**Authors:** Christoph Berendonk, Anja Rogausch, Armin Gemperli, Wolfgang Himmel

**Affiliations:** 1 Department of Assessment and Evaluation, Institute of Medical Education, University of Bern, Konsumstrasse 13, 3010 Bern, CH Switzerland; 2 Department of Health Sciences and Health Policy, University of Lucerne, Lucerne, Switzerland; 3 Swiss Paraplegic Research, Nottwil, Switzerland; 4 Department of General Practice, University Medical Center Göttingen, Göttingen, Germany

**Keywords:** clinical competence, educational measurement, clerkship, task performance and analysis, self-assessment, factor analysis, psychometrics

## Abstract

**Background:**

The mini clinical evaluation exercise (mini-CEX)—a tool used to assess student-patient encounters—is increasingly being applied as a learning device to foster clinical competencies. Although the importance of eliciting self-assessment for learning is widely acknowledged, little is known about the validity of self-assessed mini-CEX scores. The aims of this study were (1) to explore the variability of medical students’ self-assessed mini-CEX scores, and to compare them with the scores obtained from their clinical supervisors, and (2) to ascertain whether learners’ self-assessed mini-CEX scores represent a global dimension of clinical competence or discrete clinical skills.

**Methods:**

In year 4, medical students conducted one to three mini-CEX per clerkship in gynaecology, internal medicine, paediatrics, psychiatry and surgery. Students and clinical supervisors rated the students’ performance on a 10-point scale (1 = great need for improvement; 10 = little need for improvement) in the six domains history taking, physical examination, counselling, clinical judgement, organisation/efficiency and professionalism as well as in overall performance. Correlations between students’ self-ratings and ratings from clinical supervisors were calculated (Pearson’s correlation coefficient) based on averaged scores per domain and overall. To investigate the dimensionality of the mini-CEX domain scores, we performed factor analyses using linear mixed models that accounted for the multilevel structure of the data.

**Results:**

A total of 1773 mini-CEX from 164 students were analysed. Mean scores for the six domains ranged from 7.5 to 8.3 (student ratings) and from 8.8 to 9.3 (supervisor ratings). Correlations between the ratings of students and supervisors for the different domains varied between *r* = 0.29 and 0.51 (all *p* < 0.0001). Mini-CEX domain scores revealed a single-factor solution for both students’ and supervisors’ ratings, with high loadings of all six domains between 0.58 and 0.83 (students) and 0.58 and 0.84 (supervisors).

**Conclusions:**

These findings put a question mark on the validity of mini-CEX domain scores for formative purposes, as neither the scores obtained from students nor those obtained from clinical supervisors unravelled specific strengths and weaknesses of individual students’ clinical competence.

**Electronic supplementary material:**

The online version of this article (10.1186/s12909-018-1207-1) contains supplementary material, which is available to authorized users.

## Background

The mini clinical evaluation exercise (mini-CEX) is widely applied to assess clinical competencies in undergraduate and postgraduate medical education [1, 2]. In a mini-CEX, a supervisor observes a trainee during a patient encounter and rates the performance in different domains, such as history taking, physical examination, professionalism etc.

Over the years, the focus of mini-CEX has gradually shifted from *assessment of learning* to *assessment for learning* [3]. For the latter purpose, it is especially important that a mini-CEX highlights specific strengths and areas for improvement of individual trainees’ clinical performance. If, for example, a student takes a history completely and thoroughly, but is unable to come up with a reasonable differential diagnosis, he or she should receive different ratings for the two domains ‘history taking’ and ‘clinical judgement’. However, it appears that clinical supervisors do not assess the different domains separately. Several studies have demonstrated that mini-CEX domain scores correlate highly with each other [2, 4, 5]. Moreover, factor analytic studies in undergraduate [6] and postgraduate training [7] revealed that only one factor accounted for the variance in the different mini-CEX domain scores.

In contrast, there is some evidence that trainees’ self-assessed performance might capture distinct dimensions of clinical competence. Haffling and colleagues demonstrated that students who self-assessed their clinical competence in eight different domains spread their scores in a wider range compared to their educational supervisors [8]. Moreover, Braend and colleagues analysed 380 student-patient encounters and found that students were more specific and concrete in their self-evaluation compared with their supervisors [9]. Especially if the assessment serves a formative purpose, it is generally recommended to incorporate the learners’ perspective [10]. Self-assessment should stimulate learners’ reflection on their own performance and help them to identify their strengths and weaknesses [9, 11].

Although the importance of eliciting learners’ self-assessment in mini-CEX has been emphasised [3, 12], formalised self-assessment within mini-CEX is still rare. Consequently, little is known about the validity of self-assessed mini-CEX scores. According to Kane’s validity framework, the link between assessment scores and their intended interpretations is the most important step in a series of arguments [13]. In a formative setting, therefore, mini-CEX scores are valid if students and supervisors are able to draw meaningful conclusions from mini-CEX domain scores and identify specific strengths and weaknesses of the students’ performance.

The aims of this study were first, to explore the variability of students’ self-assessed mini-CEX domain scores, and to compare them with the scores obtained from their clinical supervisors; and, second, to ascertain whether students’ self-assessed mini-CEX domain scores represent—akin to supervisors’ scores—a single dimension or discrete facets of clinical competence. For this purpose, we retrospectively analysed students’ and clinical supervisors’ mini-CEX scores obtained during clerkships in undergraduate medical training.

## Methods

### Setting

During their clerkships in gynaecology, internal medicine, paediatrics, psychiatry and surgery, all 4th-year medical students at the University of Bern underwent a specified number of one to three mini-CEX per clerkship: gynaecology (2 mini-CEX), internal medicine (2 mini-CEX), paediatrics (3 mini-CEX), psychiatry (3 mini-CEX), surgery (1 mini-CEX). These clerkships can be performed in a variable order and took place at 45 different teaching hospitals affiliated with the University Medical Centre. Before the clerkships took place, interactive workshops and course material (incl. training videos) regarding the use of the mini-CEX and its formative purpose were offered to all staff responsible for the clerkship. The students received information about the aim and processes of the in-training assessment as well.

### Instruments

The mini-CEX forms were adapted from the original mini-CEX developed by the American Board of Internal Medicine [1] in order to support the formative purpose of the assessment. In contrast to the original mini-CEX, anchors for ratings were not based on labels such as ‘unsatisfactory’ to ‘superior’, but instead on ‘need for improvement’, and dedicated space was provided for narrative comments about observed strengths and areas for improvement. We have provided a detailed description of the adaptations and an example of the mini-CEX elsewhere [14].

Clinical supervisors were asked to rate the students’ performance in a directly observed student-patient interaction on a 10-point mini-CEX rating form, ranging from 1 (= great need for improvement) to 10 (= little need for improvement) in the following six domains: history taking, physical examination (for psychiatry: psychiatric status), counselling, clinical judgement, organisation/efficiency and professionalism as well as an overall impression of students’ performance. Students were asked to rate their performance on a separate form using an identical scale. Both clinical supervisors and students were asked to leave domains empty if these had not been observed or performed.

All data of this study has been collected within the regular curricular activities. According to the school’s regulation, anonymized/pseudonymised data from mini-CEX and other assessments can be used for quality assurance and research purpose. As only routinely collected, pseudonymised data were retrospectively analysed consent to participate was not possible and the study was deemed exempt from formal ethical approval according to the local regulations. Moreover, analysing the data did not affect the participants in any way.

Within this dataset, the alignment between learning needs and the learning goals [14] and score-influencing context characteristics [15] were analysed and reported separately.

### Statistical analysis

#### Variability of self- and supervisor assessments

First, we describe the students’ and supervisors’ mini-CEX ratings by domain using means and standard deviations (SDs), including a sub-analysis across the different clerkships. To analyse the correlation of the six single domain scores with each other, we calculated Pearson’s correlation coefficients (with their *p*-values) based on averaged scores per domain (separate analyses for students’ and supervisors’ assessments).

We also calculated Cronbach’s alpha as a measure of internal consistency or reliability for the 6-domain scale. To determine how each domain reflects the reliability of the scale, we also calculated a coefficient alpha after deleting each variable independently from the scale.

#### Correspondence of self- and supervisor assessments

Based on the averaged mini-CEX scores (see above), we calculated the correlations between the corresponding scores (domain and overall, respectively) obtained from student self-assessment and supervisor assessment, using Pearson’s correlation coefficients (with their *p*-values).

#### Dimensionality of self- and supervisor-assessed mini-CEX domain scores

To check whether the assessments of both students and clinical supervisors represent one global dimension or several different dimensions, we performed factor analyses.

As a first step for these analyses, we had to find a solution for the problem of missing values. Given that not all six dimensions of a mini-CEX can be necessarily observed during an individual student-patient interaction (which usually lasts for 15 min), students and clinical supervisors were instructed to rate only those dimensions of mini-CEX that were actually carried out. This policy generated a lot of missing values. To prevent the sample from shrinking too strongly, we decided to impute missing values. If one or several domains of a mini-CEX assessment were missing, we imputed the missing values with the mean of the remaining five or fewer domains. If the scores for all six dimensions were missing, we deleted the respective mini-CEX assessment. To assess the potential bias of this form of imputation, we performed a complete case analysis as sensitivity analysis.

Since our data had a multilevel structure, with assessments nested in students, clinical supervisors, clinics and specialties, we used linear mixed models to estimate the correlation matrix to be subsequently used in a factor analysis, similar to Cook et al. [7]. We considered dependence among assessments within each of the specialties, within clinics, supervisors and students and repeated assessments of the same student-supervisor pair. The exact model specification is presented with the respective SAS syntax (exemplified for clinical supervisors) in the appendix [see Additional file 1].

We performed a common factor (principal axis) analysis on the adjusted correlation matrix regarding the six subdomains, estimating initial communalities using squared multiple correlations. Finally, we repeated these analyses using principal component analysis, retaining all factors with an eigenvalue ≥1. We used varimax rotation if > 1 factors were found. All analyses were performed using SAS 9.4 for Windows (SAS Statistical Analysis System Institute, Inc., Cary, NC, USA).

## Results

### Sample and general assessment characteristics

A total of 512 clinical supervisors from 45 clinics of the University of Bern and affiliated teaching clinics were involved, resulting in a total of 1783 mini-CEX assessments for 165 fourth-year medical students (96 females). Assessments were nested in students, who were then nested in clinics (not all students had been seen by all supervisors, and nor had any single supervisor seen all students). Moreover, different students who were assigned to the same clinic were not necessarily assessed by the same supervisors, as the pool of supervisors in larger clinics is extensive. The median duration of observation was 15 min and the median duration of feedback was 5 min.

We excluded ten mini-CEX from one student with outlying low scores, resulting in a total of 1773 mini-CEX: 158 for surgery, 322 for gynaecology, 322 for internal medicine, 480 for paediatrics and 491 for psychiatry. For most of the five specialties, the required number of mini-CEX was performed by each student; the minimum was 96% for surgery, the maximum 99% for psychiatry). More than 92% of the students submitted the required number of 11 mini-CEX. While 86% (1525/1773) of the students assessed the professionalism item and 85% (1515/1773) the organisation/efficiency item, the other four items had lower rates: 76% (1344/1773) assessed clinical judgement and 74% (1305/1773) physical examination, 54% (956/1773) history taking and 27% (484/1773) counselling. The respective figures for supervisors were 86%, 82%, 72%, 77%, 58% and 31%.

### Variability of self- and supervisor assessment

**Fig. 1:**
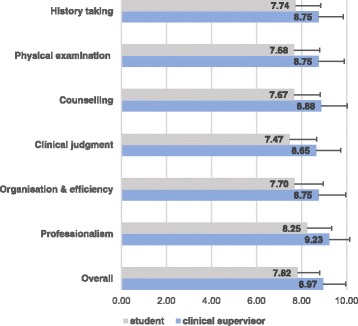
Students’ and clinical supervisors’ mini-CEX domain scores, averaged over all assessments of each student [mean ± SD]

The analyses in the following two sections are based on the students’ and supervisors’ 1773 mini-CEX assessments. Missing values were not imputed in these sections. Mean scores for overall assessment and the six domains ranged from 7.5 to 8.3 (students’ self-assessment) and from 8.8 to 9.2 (supervisors’ assessment; Fig. 1). The ceiling effect and low variability were almost the same when comparing these ratings across the different clerkships. The students’ scores ranged from 7.4 (SD 1.3) for clinical judgment in gynaecology to 8.3 (SD 1.07) for professionalism in paediatrics. The supervisors’ scores ranged from 8.3 (SD 1.4) for clinical judgement in surgery to 9.3 (SD 0.9) for professionalism in psychiatry.

All correlations between the six domains were high and statistically significant (*p* < 0.0001). Correlations ranged from 0.47 (professionalism and counselling) to 0.60 (physical examination and clinical judgement) for students, and from 0.51 (professionalism and counselling) to 0.70 (physical examination and history taking) for clinical supervisors. Although history taking and physical examination correlated somewhat higher (0.58 for students and 0.70 for supervisors), all other correlations were also at a high level. For example, the domain ‘organization’ correlated with all other domains in a range between 0.47 and 0.57 (students) and 0.52 and 0.65 (supervisors), respectively.

Cronbach’s alpha was very high for the six domains, at .86 for students’ scores and .90 for supervisors’ scores. The standardised alpha remained almost the same after removing any of the six domain variables—ranging between .83 and .85 for students and between .87 and .90 for supervisors. Each of the six variables is clearly strongly correlated with all other variables.

### Correspondence of self- and supervisor assessment

Pearson’s correlation coefficient between students’ and supervisors’ overall scores was *r* = 0.38 (*p* < 0.001). Correlations between the students’ and supervisors’ ratings for the different domains varied from *r* = 0.29 (professionalism) to 0.51 (counselling) and were all significant (*p* < 0.0001).

### Dimensionality of self- and supervisor-assessed mini-CEX domain scores

To account for the multilevel structure of the data (i.e. repeated assessments between the same student and supervisor, clinic, specialty), we performed principal components analyses on the adjusted correlation matrix estimated by the linear mixed models. We first imputed missing values in any of the six mini-CEX domains with the mean of the remaining five or fewer domains. In 73 mini-CEX from the students and in 10 mini-CEX from the supervisors, all six items were missing, meaning that imputation was not possible and the complete assessment was missing. So, the analysis for students comprised 1700 assessments, that for supervisors 1763 assessments.

Principal component analysis showed that both students’ self-assessment and supervisors’ assessments could be explained by one underlying factor only (Table 1). This factor explained 50% of the variance in student scores and 56% of the variance in supervisor scores. Factor loadings of the six domains on this single factor ranged from 0.58 to 0.83 for students’ assessment and from 0.58 to 0.84 for supervisors’ assessment.

**Table 1:** Factor loadings for mini-CEX domain scores of students’ and clinical supervisors’ assessment, following principal components analysis

Domain	Students’ assessment: factor 1 (49.7% of variance explained)	Clinical supervisors’ assessment: factor 1 (56.2% of variance explained)
History taking	0.78	0.80
Physical Examination	0.73	0.76
Counselling	0.83	0.84
Clinical judgment	0.67	0.72
Organisation & Efficiency	0.69	0.73
Professionalism	0.58	0.58

We repeated the factor analysis for a complete case sensitivity analysis, excluding all mini-CEX with one or more missing values. This reduced the sample of mini-CEX from *n* = 1700 to 171 in the case of students and from *n* = 1763 to 222 in the case of supervisors. Unsurprisingly, the loading of the six items and the explained variance were lower, but again, only one factor emerged in both cases (data not shown).

## Discussion

The variability of students’ and clinical supervisors’ mini-CEX domain scores obtained from different clerkships in undergraduate training was rather low. Both of these scores showed a notable ceiling effect, which was particularly pronounced among clinical supervisors. The correlation between the students’ and supervisors’ scores was moderate to fair. More importantly, all six domain scores correlated consistently high, even those dimensions that had, at first glance, little in common such as counselling and organization. Consequently, factor analysis of students’ and supervisors’ scores revealed a single-factor solution.

Range restriction and high ratings for mini-CEX scores have been described in other studies from undergraduate [16, 17] as well as postgraduate education [18, 19]. This range restriction of real-life performance assessment stands in contrast to the wide range of scores attributed to performance assessment in simulated/experimental settings, where there is no professional relationship between trainees and assessors [5, 20, 21]. Such grade inflation in apprenticeship types of assessment may be due to the supervisors’ double role [22]: If clinical supervisors have to decide between their role as a coach (assisting the trainee in improving their clinical skills) and their role as a judge (rating the performance of the trainee), they usually choose the role of the coach [23].

At first glance, students’ self-assessed scores seem to be somewhat more realistic and less prone to grade inflation compared to scores from their supervisors. However, self-assessment may also be ‘strategic’ and influenced by the social context and direct interaction with the teacher. This is unsurprising, as self-assessment is best characterised by ‘multiple tensions arising from complex interactions among competing internal and external data and multiple influencing conditions’ [24]. In other words, self-assessment is conducted against the background of diverse, mutually interacting factors: purpose of the assessment (whereby the officially declared goal does not have to be congruent with the personally defined goal of the individual student), belief about one’s self-efficacy [25] and the specific characteristics of the context in which the self-assessment takes place [26].

In general, self-assessments should never stand alone and should always be accompanied by feedback from supervisors ― not least because students tend to over- or underestimate their performance [27]. In our study, the correlations between students’ and supervisors’ mini-CEX scores were moderate to fair. These findings are in line with several other studies ― summarised in a systematic review by Colthart et al. ― indicating that practical skills may be better measured through self-assessment than through knowledge-based activities [28]. This correspondence between students and supervisors is reassuring and might be a starting point for discussions (feedback) about individual students’ clinical performance and how it can be improved in the future.

Factor analysis of clinical supervisors’ mini-CEX domain scores revealed one single underlying factor. This finding corroborates the results of Cook [7], who found a single factor for trainers’ mini-CEX domain scores of internal medicine residents. Our results thus add to the understanding of mini-CEX scores by replicating Cook’s findings in a different setting (Europe vs. North America), at a different stage of education (undergraduate vs. postgraduate education) and applying different types of scale (10- vs. 5-point scale).

More importantly, factor analysis of students’ self-assessed mini-CEX domain scores also revealed a single-factor solution. In other words, not only supervisors’ but also students’ mini-CEX domain scores measure a single global dimension of clinical competence. The difficulty of treating an individual performance as a compound of separate qualities and of assigning an individual score to each of these qualities is known as the ‘halo’ effect. This effect was first described by Thorndike almost a century ago [29]. Results of experimental studies suggest that halo effects operate in social interaction as well [30]. These findings highlight once again the fundamental influence of social factors in human judgement [22].

Moreover, other studies from psychology demonstrate that decision makers who feel accountable for their actions exhibit greater analytic complexity [31]. It can be argued that in our setting, with its formative purpose of the assessment, neither students nor clinical supervisors attributed a great deal of importance to the scores assigned. Factor analytic studies of performance assessment with a summative purpose, in contrast, demonstrate a two-dimensional model [32, 33]. In other words, a lack of accountability may be an additional reason why students and supervisors alike assessed students’ performance by only one dimension.

### Limitations and strengths of the study

More rigorous faculty and student training (in the use of the mini-CEX) might have led to a somewhat different outcome. However, faculty training seems to have no relevant impact on factor structure [7] or reliability [34]. The complex, multifaceted nature of self-assessment makes it implausible that a limited intervention would substantially change the self-assessment behaviour of students either [25].

Part of the correlation between students’ self-assessed scores and scores from supervisors might be due to prior (oral) exchange of evaluation before filling in the assessment form. We have no data on / insight into how the encounter actually took place. However, students and supervisors were instructed to fill in the assessment form first, and only after that to start the (verbal) feedback. Moreover, a reasonably good correlation of self-assessment with observed measures of practical skills has also been described by others [35–38].

Due to a rather high number of missing values, at least in some mini-CEX domains, we imputed missing data. We had the choice between two alternatives—both entailing advantages and disadvantages:

(i) Following the idea of an ‘overall impression’ of an examination, one could impute the missing value with the mean of the remaining five or fewer domains of the mini-CEX. Of course, if all six domains are missing, no imputation can be performed and the complete assessment is missing. This imputation has the advantage that it reflects the current state of a student’s knowledge and skills in the respective medical area. The disadvantage is that this imputation supports the supposed tendency of the mini-CEX scores to represent only one underlying factor.
(ii) In another form of imputation, one could replace a missing value with the average of the same person in the same domain from other non-missing mini-CEX examinations. If, for example, a student or the supervisor had not filled in a score for history taking in a paediatric examination, one could impute this missing value with the student’s mean of all history-taking scores from his or her other examinations. While this approach would not necessarily support the one-factor hypothesis, this imputation procedure would be in conflict with the well-established concept of ‘content specificity’ of competence [39]. Performance in one area does not predict performance in other areas very well [40]. Moreover, such an imputation procedure would not allow the consideration of changes over time. For all of these reasons, we decided on the first alternative. This approach was validated with a complete case analysis as sensitivity analysis.

The strength of this study is the large sample size, including several specialties and teaching clinics. However, we have to consider that each student contributed to more than one assessment and the students were nested within specialties, clinics and supervisors. In such cases, statistical dependency may occur. The multilevel design of the study helped to avoid considering the assessments as independent and violating the independence assumption of conventional statistical methods. At first glance, the high number of missing values in certain mini-CEX domains might be seen as a weakness of our study. However, an assessment of all six domains in every 15-min student-patient encounter would not have been credible. Moreover, the distribution of scored (and missing) domains is meaningful, as the generic domains of ‘organisation/efficiency’ and ‘professionalism’ were assessed in the vast majority of all mini-CEX. In contrast, students and clinical supervisors had to set a focus on either ‘history taking’, ‘physical examination’ or ‘counselling’ in the 15-min encounter. Unsurprisingly, ‘counselling’ was the domain with the highest number of missing values: It is a skill that is often not yet mastered and therefore not implemented by medical students in their first clerkships. We therefore argue, much to the contrary, that the number of missing values is convincing evidence that the students and clinical supervisors used the mini-CEX carefully and conscientiously.

### Implications for practice

Our results have implications for the utility of mini-CEX scores in undergraduate medical education. Neither the assessment from students nor that from supervisors seems to differentiate between separate dimensions of medical students’ clinical competence (i.e. history taking, physical examination, clinical judgement). The fact that mini-CEX domain scores cannot differentiate between separate aspects of clinical competence and that variance in mini-CEX scores arises to a far greater extent from supervisors than from students [19, 41] threatens the validity of mini-CEX scores. Cook and colleagues concluded that such findings render mini-CEX domain scores inadequate for moderate- or high-stakes summative assessment [7]. If Kane’s framework is used to construct an argument to support the intended interpretations of formative mini-CEX domain scores—to detect specific strengths and weaknesses of individual students’ clinical competence—it becomes evident that such scores have little validity. As validity is not a property of an instrument per se but rather of the instrument scores to be used for a specific purpose [42], we would like to extend Cook’s statement insofar as mini-CEX domain scores are of limited value in formative assessment as well.

These findings do not necessarily contradict the importance of mini-CEX in formative in-training assessment. However, the value of directly observed student-patient interaction lies not in (the inherently flawed) scores but rather in the rich narrative feedback that stimulates a meaningful discussion between students and clinical supervisors [3, 22].

## Conclusions

Students’ self-assessed mini-CEX domain scores as well as the scores obtained from their clinical supervisors measure a single global dimension of medical students’ clinical competence. This finding puts a question mark on the utility of mini-CEX domain scores for formative purposes, as these scores do not unravel specific strengths and weaknesses of individual students’ clinical competence.

## Additional file

Additional file 1: Appendix. SAS code for the multilevel factor analysis, exemplified for clinical supervisors. (DOCX 19 kb)

## Abbreviations

mini-CEX: Mini clinical evaluation exercise
SAS: Statistical Analysis System
