# Supplementary material for: Variability and dimensionality of students’ and supervisors’ mini-CEX scores in undergraduate medical clerkships – a multilevel factor analysis
Source: BMC Med Educ. 2018 May 8;18:100. doi: 10.1186/s12909-018-1207-1 (PMC5941409; doi:10.1186/s12909-018-1207-1)
Supplement: Supplementary file 1 — Appendix. SAS code for the multilevel factor analysis, exemplified for clinical supervisors. (DOCX 19 kb) [file 12909_2018_1207_MOESM1_ESM.docx]

Appendix

SAS code for the multilevel factor analysis, exemplified for clinical supervisors

/* Turn data table to vertical*/

**data** iml3;

set iml2;

array e_itm (**6**) e_histtake_n--e_profess_n;

do item=**1** to **6**;

rating=e_itm(item); output;

end;

**run**;

**proc** **sort** data=iml3;

by item;

**run**;

**proc** **sort** data=iml3 out=iml4;

by student_id expert_id rep_id;

**run**;

/* generate unique identifier for combination of student-expert-repetition */

**data** iml4;

set iml4;

by student_id expert_id rep_id;

if first.rep_id then id+**1**;

**run**;

**data** iml5;

set iml4;

if e_missing > **5** then delete;

**run**;

/* proc mixed */

/* controlled for student, expert, specialty and clinic effects */

ods output RCorr=Corr;

**proc** **mixed** data=iml4;

class item specialty_num n_stud_gruppen3 clinic expert_id student_id expert_gender student_gender rep_id;

model rating = item / solution;

random specialty_num;

random n_stud_gruppen3;

random expert_id;

random student_id;

repeated item / subject=student_id*expert_id*rep_id type=un rcorr;

**run**;

/* pack covariance, mean, standard deviation and estimated effective sample size into one dataset */

/* Mean, Stddev */

ods output summary=sum;

**proc** **means** data=iml3 mean stddev;

var rating;

by item;

**run**;

**proc** **transpose** data=sum out=sum2(drop=_name_ _label_) prefix=Col;

var rating_mean rating_stddev;

id item;

**run**;

/* Estimate effective sample size */

ods output summary=sum;

**proc** **means** data=iml3 N;

var item;

by item;

where rep_id=**1**;

**run**;

**proc** **transpose** data=sum out=sum3(drop=_name_ _label_) prefix=Col;

var item_n;

id item;

**run**;

/* Combine */

**data** corrmatrix(drop=row index type=corr);

set sum2(in=dA) sum3(in=dB) corr(in=dC);

if dA then do;

if _N_=**1** then _Type_='MEAN'; else _Type_='STD';

end;

if dB then _type_='N';

if dC then do;

_type_='CORR';

_name_='Col'||compress(put(row,**2.**));

end;

**run**;

ods select all;

**proc** **factor** data=corrmatrix priors=smc scree rotate=varimax;

**run**;
